# From matching to facilitation: Reframing AI’s role in clinical trial enrollment

**DOI:** 10.1016/j.patter.2026.101591

**Published:** 2026-07-10

**Authors:** Adar Yaacov, Einat Levi, Nir Peled

**Affiliations:** 1 Helmsley Cancer Center, Shaare Zedek Medical Center, Jerusalem, Israel; 2 Faculty of Medicine, The Hebrew University of Jerusalem, Jerusalem, Israel

**Keywords:** clinical trial enrollment, artificial intelligence, trial matching

## Abstract

Yaacov, Levi, and Peled argue that AI-powered clinical trial matching has achieved near-expert technical accuracy yet fails to increase patient enrollment because the dominant bottleneck is systemic—involving logistics, workflow misalignment, and consent burden—not informational. They propose reframing AI’s role from matching engine to trial facilitation system.

## Main text

Artificial intelligence (AI) can now match cancer patients to clinical trials with near-expert accuracy. In a rigorous randomized trial at one of the world’s leading cancer centers, this capability produced no additional enrollments.^1^ This is not a failure of AI. It is a signal that matching accuracy, while necessary, is not sufficient and that the remaining constraints on enrollment lie elsewhere in the clinical ecosystem.

### Matching accuracy is no longer a primary bottleneck

The technical challenge of identifying which patients might be eligible for which trials has seen remarkable progress. TrialGPT, a large language model framework developed at the NIH, achieves 87.3% accuracy in criterion-level patient-trial matching, approaching expert-clinician performance, and reduces screening time by over 40%.^2^ MatchMiner, an open-source platform at Dana-Farber Cancer Institute, algorithmically matches patient genomic and clinical data to precision medicine trial eligibility and has supported over 400 patient enrollments.^3^ Commercial platforms including Tempus and Triomics have attracted substantial investment around similar capabilities. These are genuine achievements. Natural language processing now parses the unstructured complexity of clinical notes, radiology reports, and pathology results to surface trial opportunities that would otherwise be invisible. While residual false-positives and false-negatives and untested generalizability remain, the marginal gap between current systems and perfect matching is narrowing and unlikely to be what is holding enrollment rates.

Yet fewer than 5% of adult cancer patients globally enroll in clinical trials, a figure that has barely changed in two decades despite this technical progress.^4^ The oncology trial pipeline continues to expand—there are more than 2,000 unique clinical-stage assets competing for patients—and the gap between the demand for trial participants and actual enrollment widens each year. This suggests that improving matching accuracy alone will not close the gap and that the remaining barriers lie elsewhere.

### The puzzle

In April 2025, Mazor and colleagues at Dana-Farber published the results of a single-center randomized trial involving 20,707 patients with genomically characterized solid tumors.^1^ An AI model analyzed unstructured clinical notes to detect disease progression (the moment a patient would most likely need a new treatment line) and then triggered notifications to treating oncologists about genomically matched therapeutic trials. The trial was conducted at a center with a research-rich culture and deep infrastructure. The design was elegant: the right information, to the right physician, at the right time.

The result was null. Patients whose oncologists received AI-triggered trial notifications were no more likely to enroll in a clinical trial than controls. The AI correctly identified patients with progressive disease. The notifications reached the intended physicians. The genomic matches were valid. Nothing changed.

This finding deserves more than a passing acknowledgment as a negative trial. It is, in effect, a diagnostic test performed on the field itself. While Mazor and colleagues tested a specific delivery mechanism of physician notifications, and cannot rule out alternative information bottlenecks, the study nonetheless narrows the hypothesis space: closing the matching-accuracy gap alone, however elegantly, is unlikely to move enrollment significantly. The systematic barriers to clinical trial participation—structural, clinical, and attitudinal—have been extensively documented,^5^ and decades of interventions have failed to meaningfully shift enrollment rates. Perhaps this is because many of those interventions, like AI-based matching, have addressed one dimension of a multidimensional problem.

### Why notifications alone fall short: A view from the clinic floor

We write from direct experience on both sides of this problem. We operate a dedicated AI unit within a comprehensive cancer center that manages over 120 active clinical trials and treats approximately 2,000 new patients annually, serving a diverse population of approximately 1.5 million. One of us (N.P.) has led clinical trial recruitment in oncology for over two decades, overseeing the enrollment of thousands of patients across institutional, national, and multinational trials. Another (A.Y.) develops and deploys AI-based tools for clinical trial matching and recruitment within the same department. This dual vantage point—building AI matching systems while simultaneously recruiting patients through traditional clinical pathways—has shown us that technically sound matches frequently fail to translate into enrolled patients, not because the algorithm erred, but because the clinical ecosystem surrounding the match was not designed to act on it. From this experience, we observe five systemic barriers that matching algorithms, however accurate, do not address on their own.

**Figure 1:**
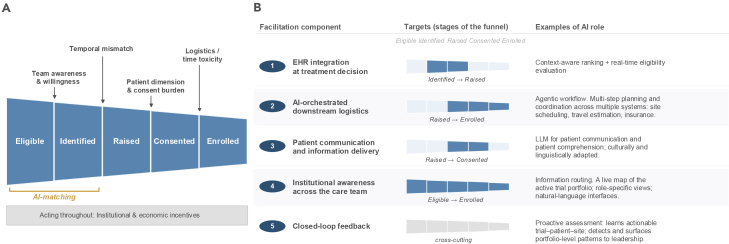
The clinical trial enrollment funnel and facilitation (A) Five-stage funnel from eligibility to enrollment, with an example of a barrier driving each transition annotated. (B) Facilitation: five components designed to mitigate attrition through advanced AI capabilities. Each component is mapped to the specific funnel transition it targets, alongside examples of its potential AI role.

Enrollment unfolds as a funnel rather than a single decision: an eligible patient must be identified, the opportunity must be raised within a clinical encounter, the patient must consent, logistical requirements of the study must be met, and, once enrolled, the patient must be retained on study. Matching addresses only the first of these stages, and each subsequent stage imposes its own attrition (Figure 1A).

#### Team awareness and willingness

The most important determinant of trial accrual is not algorithmic precision—it is the awareness and willingness of the entire clinical team to prioritize research alongside care. Trial recruitment is a team behavior involving medical doctors, nurses, study coordinators, and more. When this collective orientation exists, patients are identified through the natural rhythm of clinical work. When it is absent, even accurate AI notifications can land in a vacuum. An automated alert is most powerful when it reaches a team that is already oriented toward research. No algorithm can substitute for a clinical culture that treats research as integral to patient care. AI can, however, support and amplify this culture.

#### The patient dimension

Patients who are eligible on paper may decline for reasons invisible to any algorithm: caregiver fatigue, fear of randomization, preference for the certainty of standard treatment, or end-of-life priorities that favor quality of remaining time over experimental possibility. These decisions are further shaped by cultural context. In some societies, patients and families gravitate toward novel, cutting-edge interventions; in others, trust is reserved for treatments with an established track record. Beliefs about fate and agency influence the calculus as well and might be overlooked. Serving a culturally diverse population, we often see how cultures that emphasize acceptance of one’s destiny may view experimental treatment differently from those that prize active intervention against illness. Moreover, in low- and middle-income settings where modern standard-of-care options are limited or unavailable, the primary motivation for trial participation may be access to otherwise inaccessible treatments rather than the research question itself.

#### The consent and logistics burden

Clinical trial enrollment is not a prescription. It is a process. The informed consent discussion alone typically requires 60 to 90 min—time that competes with a clinic schedule where each patient slot is 15 to 20 min. Recruiting over 2,300 patients across eight countries for the I^3^LUNG study^6^ has made this acutely visible to us: even motivated investigators at well-resourced sites face logistical friction that no matching algorithm anticipates. Coordinating multi-omics sample collection across pathology, radiology, and genomics departments consumes weeks of coordinator effort per patient. Regulatory requirements differ across countries. Study-specific visit schedules diverge from routine care pathways. For patients, the burden compounds further: additional blood draws, imaging at specified intervals, travel to trial sites, insurance pre-authorizations, and companion requirements. This is what recent literature has termed “time toxicity”^7^—the cumulative non-medical burden of trial participation that falls disproportionately on patients who are already ill. No matching algorithm accounts for it because it is not a matching problem.

#### Temporal mismatch

A trial match is only actionable at the precise moment a patient needs a new treatment line (including 1^st^ line). That moment is embedded in a turbulent clinical trajectory where performance status is declining, a prior regimen is failing, and the patient is sitting across from you, anxious, often accompanied by an overwhelmed family. The decision to change treatment unfolds over a 15-to-20-min clinic visit in which the oncologist is simultaneously weighing three or four therapeutic options, managing toxicities, and navigating the patient’s emotional state. A trial enrollment rarely results from a notification alone—it emerges organically from the clinical encounter itself, when the physician recognizes in real time that this patient, at this moment, might benefit from an investigational approach.

#### Institutional and economic incentives

Cutting across the barriers above, institutional pressures and economic incentives, at both the organizational level (accrual targets and sponsor reimbursement) and the individual level (investigator research funding and academic credit), shape clinical trial engagement and enrollment.

### A broader pattern

The clinical trial-matching puzzle illustrates a broader pattern in clinical AI: the tendency to optimize where measurement is tractable rather than where impact is greatest. Matching is computationally well-defined—structured inputs, structured outputs, measurable accuracy on benchmarks. The actual enrollment problem is messy, multi-stakeholder, and deeply context dependent, precisely the kind of problem that resists clean algorithmic formulation.

This pattern extends beyond trial matching. Drug response prediction models achieve impressive discriminative performance on cell-line data but struggle to transfer to patient outcomes. Radiology AI tools flag abnormalities with high sensitivity but frequently fail to change diagnostic or therapeutic pathways. The common thread is not that these tools lack value, as they clearly do, but that technical performance on an isolated component of a clinical workflow does not automatically translate to impact on the workflow’s ultimate outcome. The surrounding system must be ready to act on the output.^8^

### From matching to facilitation

If matching alone is insufficient to move enrollment, what else is needed? Our experience suggests a conceptual reframe: from trial matching to trial facilitation (Figure 1B).

First, AI should be contextually integrated into the treatment-planning workflow rather than delivered as a separate notification. Trial options should appear alongside standard-of-care alternatives within the electronic health record at the moment the oncologist is already making a treatment decision—with real-time eligibility screening that includes clinical parameters, not only genomic ones. This is a context-aware recommendation challenge: ranking trial options by fit within an existing clinical decision interface.

Second, AI should orchestrate the downstream logistics. If a patient is potentially eligible, can the system automatically verify scheduling availability at trial sites, estimate travel requirements, flag insurance complexities, and prepare a referral package before the oncologist decides whether to raise the option? An AI-based system that compressed this process to minutes would address the friction where it actually accumulates. This is an agentic architecture challenge.

Third, AI should reduce the consent burden. Patient-facing tools could generate readable trial summaries, expected visit schedules, and plain-language comparisons between the trial and standard care, transforming the consent process from a 90-min information transfer into a prepared, informed conversation. This is among the most immediate clinical applications for large language models as patient-communication tools.

Fourth, facilitation systems should build institutional awareness. Rather than notifying only the treating oncologist, AI tools should surface the active trial portfolio to the entire care team—nurses, study coordinators, and residents—so that potential candidates can be flagged during routine clinical interactions such as triage, rounds, and follow-up calls. The goal is a shared research culture supported by continuous, embedded visibility. Computationally, this is a multi-stakeholder information-routing problem, requiring models that target different roles at different points in the care pathway.

Fifth, facilitation systems should maintain feedback loops, tracking why offers were declined and incorporating that knowledge into future recommendations. For example, if most declines at a given site are logistics related, the system should deprioritize distant trials and negotiate satellite enrollment options. If consent complexity is the primary barrier for a particular trial, the system should flag this to the sponsor as an accrual risk. This closed-loop architecture—match, facilitate, track, learn—represents a fundamentally different philosophy from “match and notify.” Computationally, it reframes trial enrollment as a contextual bandit problem: the system must learn which trial-patient-site combinations are actionable, not merely eligible, and update its policy from real-world outcomes.

### Conclusion

The clinical trial-matching puzzle teaches a lesson worth internalizing: that the most consequential barriers in oncology trial enrollment are only in part computational ones. They are human, logistical, and systemic. As clinicians who build and implement AI trial-matching tools, we believe deeply in their potential, but we also see, daily, that technically valid matches require far more than accuracy to become enrolled patients. The algorithms work. What is missing is the surrounding infrastructure to act on their output. The next generation of AI for clinical trials should not aim solely to match patients more accurately—that capability is maturing rapidly. It should aim to make enrollment easy. When AI moves from recommending trials to actively reducing the friction of participating in them, the field will begin to see the enrollment gains that matching accuracy alone has not delivered.

## Declaration of interests

A.Y. is an inventor of the patent “Machine learning identification of mutational signatures” and reports fees from Protica Bio. N.P. reports advisor and honorarium from and research with AstraZeneca, Bayer, Boehringer Ingelheim, Bristol-Myers Squibb, Eli Lilly, Imagine, Guardant360, Imagene, Genesis, Merck, MSD, Novartis, Pfizer, Roche, Rhenium, and Takeda.

## Declaration of generative AI and AI-assisted technologies in the writing process

During the preparation of this work the authors used Claude Opus 4.6 to improve language, refine phrasing, and assist with revisions of the manuscript text. After using this tool, the authors reviewed and edited the content as needed and take full responsibility for the content of the published article.
